# Complexity and Robustness of Public–Private Partnership Networks

**DOI:** 10.3390/e28010122

**Published:** 2026-01-20

**Authors:** Na Zhao, Xiongfei Jiang, Ling Bai

**Affiliations:** College of Finance and Information, Ningbo University of Finance and Economics, Ningbo 315175, China

**Keywords:** public–private partnership, complex network, robustness, geographic structure

## Abstract

Public–private partnership (PPP) has been increasingly imported to deliver infrastructure and public services around the world. As an emerging public procurement mode, PPP has drawn considerable attention both from academy and industry. We construct a PPP shareholder network of China and analyze its topological complexity, robustness, and geographic structure. We find that the PPP shareholder network exhibits small-world behavior and a heavy-tailed degree distribution. Using multiple centrality measures, we investigate the network robustness under various attack strategies. The results show that the targeted attack destroys the network more efficiently than the random attack, especially the degree-based and betweenness-based attacks. For geographic topology, it exhibits a hierarchical spatial structure in which Beijing is the central hub and provincial capitals are regional centers. Our research has significant implications for policy-making to improve supervision for enterprises involved in PPP projects.

## 1. Introduction

PPPs are contractual partnerships between actors in public and private sectors. For several decades, governments worldwide have increasingly imported PPP to improve the infrastructure, such as for sewage treatment and highway construction [1,2,3,4]. The PPP system runs through contractual partnerships between institutions in the public and private sectors, such as design–finance–build–maintain and build–operate–transfer [5]. During the partnership, the public and private sectors share risks and benefits in order to achieve common goals.

At present, much of the literature focuses on the micro level of PPP mode, such as the contract governance [6,7], relationships among stakeholders [8,9] and the risk transmission mechanisms of single projects [10,11], while the macro level of PPP mode including network structure across different PPP projects requires further investigation.

PPP projects involve multiple participants who form a close-knit relationship network within a single project. If one participant is involved in multiple projects, this creates links between different projects. These participants collaborate across different projects, weaving a larger industry-wide network [12,13]. This network describes information flows and resource transactions between participants to achieve mutual gains [14,15]. However, PPP projects are subject to multiple risks, such as withdrawal, default or bankruptcy of shareholder, and sudden changes in policies or market fluctuations. If one shareholder withdraws, it may affect the smooth progress of multiple PPP projects. Furthermore, if multiple shareholders withdraw, this may affect the stability of the entire PPP network. These risks can interact with each other [16], and propagate through the project network [10].

The network theory has been introduced to various fields, from financial markets [17] to biomedical systems [18,19] and social systems [20,21]. Especially, economic activities are modeled as complex networks, such as microfinance [22], labor flow [23], and supply chains [24,25]. Several universal properties, including small-world and scale-free, are well studied in those networks [18,19,26]. In particular, with recent developments in econophysics, there is a growing recognition that connections between institutions may generate collective emergent behaviors, such as robustness and systemic risk. Although robustness and resilience are universal properties of different complex systems, these concepts can be approached from unique perspectives. The rich interdisciplinary research has generated various descriptions of robustness and resilience due to the specific objectives of fields, such as the resilience of smart grids [27] and intelligent transportation systems [28], the regime shift in coral reefs [29] and tropical forests [30], and the resilience of supply chains against the COVID-19 pandemic [24].

Different network structures exhibit significant differences in robustness. Homogeneous networks tend to respond similarly to different attacks [31]. In contrast, heterogeneous networks, such as scale-free networks, demonstrate different responses to the random and targeted attacks. Scale-free networks show strong robustness to the random attack, but are highly vulnerable to the targeted attack [26]. The connectivity and robustness of the PPP shareholder network are closely related to the information exchange and financing of the PPP system, which significantly affects the infrastructure. Although knowledge of PPP expands rapidly, the function of shareholder network maintaining the PPP system remains an open question [32,33].

Based on the PPP project dataset, we construct an undirected graph named the PPP shareholder network (P3SN). This network not only describes the collaborations between shareholders belonging to the same PPP project, but also presents the connections among those across different PPP projects. The key contributions and innovations of this study are outlined below:

(1) This study shifts the research perspective of PPP mode from the micro to the macro level by constructing a cross-project shareholder collaboration network. It is different from the traditional literature that focuses on PPP networks within the same industry. This approach more comprehensively reveals the overall structure and connections of the PPP system in China.

(2) Using complex network theory and network metrics, this paper systematically analyzes the structural complexity of the P3SN. We identify key nodes of P3SN by comprehensively applying various centralities and evaluate robustness of the P3SN under different attack strategies. Furthermore, the relationship between network robustness and topology is investigated. The results indicate that the P3SN is a small-world network with a heavy-tailed degree distribution.

(3) We project the P3SN onto a map to visually present the spatial structure of the shareholder collaboration network, providing new perspective for understanding the regional distribution of shareholder collaborations in PPP projects. P3SN map exhibits hierarchical structure with Beijing as the central hub and provincial capitals as regional centers. These findings provide important insights for identifying key shareholders in PPP projects.

## 2. Centrality and Robustness Measures

An important research topic is how to maximally destroy a network by removing a specified number of nodes. Nodes removed during this process are usually referred to as “key nodes”. In this paper, for the P3SN with a node number of *N*, the “key nodes” are determined by one of four centrality measures, which are degree, entropy, betweenness, and closeness. In this study, four indicators are selected to evaluate the robustness of shareholder network, including the relative size of the giant connected component (R), average path length (L), average clustering coefficient (C), and network efficiency (E).

### 2.1. Degree Centrality

Degree centrality is defined as the number of neighbors directly connected to the node [34,35]. It is the simplest and most intuitive centrality metric for measuring node importance. The adjacency matrix *A* is defined as

$$A_{ij}=\left\{\begin{array}{cc} 1 & \;,\;\mathrm{if}\;\mathrm{there}\;\mathrm{is}\;a\;\mathrm{link}\;\mathrm{between}\;\mathrm{the}\;i-\mathrm{th}\;\mathrm{node}\;\mathrm{and}\;\mathrm{the}\;j-\mathrm{th}\;\mathrm{node}\\ 0 & \;,\;\mathrm{otherwise}. \end{array}\right.$$

Thus, the degree centrality $k_{i}$ of a node *i* is defined as $k_{i}=\sum_{j=1}^{n}A_{ij}$, where *n* is the number of direct neighbors of node *i*.

### 2.2. Entropy Centrality

**Figure 1:**
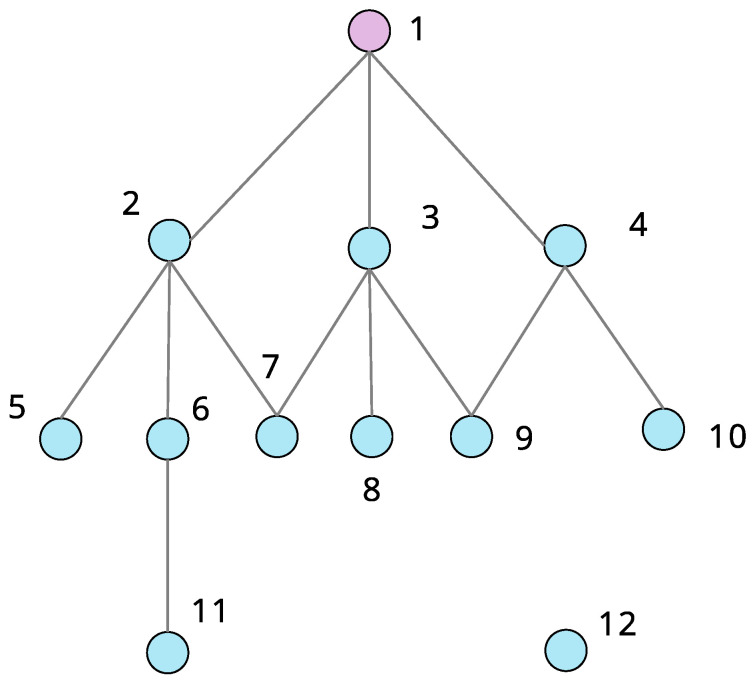
Illustration of the sequence of information collected by a node in a network [36]. The friends of node 1 are nodes 2, 3, and 4. The friends of friends of node 1 are nodes 5, 6, 7, 7, 8, 9, 9 and 10. The information sequence is 2, 3, 4, 5, 6, 7, 7, 8, 9, 9, 10, with a size of 11, and the frequencies of nodes in information sequence are $q_{1}=q_{2}=q_{3}=q_{4}=q_{5}=q_{6}=q_{8}=q_{10}=\frac{1}{11}$, $q_{7}=q_{9}=\frac{2}{11}$, and $q_{1}=q_{11}=q_{12}=0$. Thus, the information entropy of node 1 can be calculated based on the definition of entropy centrality in Section 2.2.

We adopt information entropy as a centrality to quantify the importance of a node [36]. The information entropy for node *i* is defined as

$$S\left(i\right)=-\sum_{j=1}^{n}q_{j}logq_{j},$$

where $q_{j}$ is the frequency of node *j* in the information sequence, and *n* is the number of nodes in information sequence, as shown in Figure 1. Thus, it implies that the information entropy of a node is determined by its friends and friends of friends.

Information entropy is used to measure the quantity and diversity of information. In social networks, maximizing friendship diversity by maximizing information entropy is essential to acquire optimal information. The entropy centrality used in this paper is different from other centrality metrics, such as betweenness and closeness. It is generated from local information, whereas betweenness centrality and closeness centrality require global knowledge of the network. Furthermore, it is also different from degree centrality, because it does not simply evaluate the importance of a node by the number of its friends. Nodes with high entropy are prone to propagating information throughout the network. Their removal is more likely to undermine the network robustness. Thus, the entropy centrality serves as an important indicator for identifying key nodes [37].

### 2.3. Betweenness Centrality

In a network, information is often transmitted via the shortest paths [38]. The greater the number of shortest paths pass through a node, the more information passes through it. The shortest path between these two nodes is called a geodesic path. The number of geodesic paths between all pairs of nodes that pass through it is defined as the betweenness of the node [25]. Nodes with high betweenness control the flow of information between other nodes, which has significant influence within the network. Nodes with high betweenness centrality act as a “bridge” between other nodes [34]. The betweenness centrality is expressed as

$$b_{i}=\sum_{u,v}\frac{n_{i}\left(u,v\right)}{g(u,v)},$$

where $n_{i}\left(u,v\right)$ is the number of the geodesic paths from node *u* to *v* passing through node *i*, and the g(u,v) is the total number of geodesic paths from node *u* to *v*.

### 2.4. Closeness Centrality

Closeness centrality provides a different measure of centrality, which quantifies the average distance paths from a node to other nodes [38]. Assuming d(i,j) denotes the length of the geodesic path from node *i* and node *j*, the average geodesic distance between node *i* and all other nodes is

$$l_{i}=\frac{1}{N-1}\sum_{j\neq i}d\left(i,j\right).$$

The shorter the geodesic distances from a node to other nodes, the smaller its average geodesic distance and the more easily it can access information from them. Closeness centrality is formally defined as the reciprocal of $l_{i}$ by

$$CL_{i}=\frac{1}{l_{i}}=\frac{N-1}{\sum_{j\neq i}d\left(i,j\right)}.$$

### 2.5. Relative Size of Giant Connected Component

The relative size of giant connected component serves as a primary indicator of structural robustness of network, quantifying the proportion of nodes maintaining connectivity after the network is disturbed or impacted. In a initial network with node number of *N*, after removing specified number of nodes, $N^{\prime }$ is the number of nodes in the new giant connected component. Thus, the relative size of the giant connected component for this network is $R=N^{\prime }/N$, which intuitively reflects the overall connectivity of the network.

### 2.6. Average Path Length

The average path length describes the mean geodesic path length over all nodes pairs in the network, which is defined as

$$L=\frac{1}{N(N-1)}\sum_{i<j}d\left(i,j\right).$$

The shorter the average path length of a network, the faster information can propagate throughout the entire network.

### 2.7. Clustering Coefficient

Clustering coefficient quantifies the density of triangles within a network [38]. This paper uses the average of the local clustering coefficient as the clustering coefficient of the whole network. The local coefficient of node *i* is given by the fraction of pairs of its neighbors which are mutually connected as follows:

$$C_{i}=\frac{\mathrm{count}\;\mathrm{of}\;\mathrm{neighbor}\;\mathrm{pairs}\;\mathrm{of}\;\mathrm{node}\;i\;\mathrm{that}\;\mathrm{are}\;\mathrm{directly}\;\mathrm{connected}\;\mathrm{to}\;\mathrm{each}\;\mathrm{other}}{\;\mathrm{total}\;\mathrm{number}\;\mathrm{of}\;\mathrm{neighbor}\;\mathrm{pairs}\;\mathrm{of}\;\mathrm{node}\;i}.$$

Then, the clustering coefficient of the whole network is

$$C=\frac{1}{N}\sum_{i=1}C_{i}.$$

Essentially, it assesses the probability that two neighbors of a given node are connected to each other. A high clustering coefficient indicates a significant node aggregation effect within the network, which means that nodes tend to form tight clusters.

### 2.8. Network Efficiency

Network efficiency is used to measure the efficiency of information propagation in a network [39]. If there is no path between node *i* and *j*, $d_{ij}=\infty$. Thus, network efficiency measures the efficiency of information propagation, which is defined as

$$E=\frac{1}{N(N-1)}\sum_{i\neq j}\frac{1}{d_{\left(i,j\right)}}.$$

## 3. Data

We collected the data from the “Wind Financial Database” from 2012 to 2019, containing 5187 PPP programs. After excluding programs containing individual, overseas, and consortium shareholders, 4933 programs remain. In this study, individuals, overseas, and consortium shareholders are excluded for two primary reasons. Firstly, robustness tests demonstrate that the inclusion or exclusion of these shareholders has a minimal impact on the composition of key node sets. Secondly, the subsequent part of the paper constructs a shareholder network based on geographic locations within China. As individual, overseas, and consortium shareholders lack clearly defined location information within China, they were excluded.

**Figure 2:**
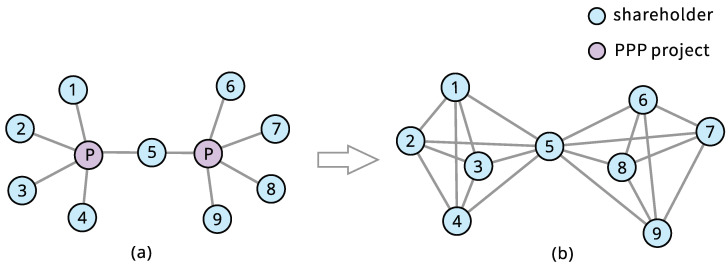
Demonstration of the P3SN generation process. (**a**) The network of shareholders and PPP projects. (**b**) The network of shareholders.

**Figure 3:**
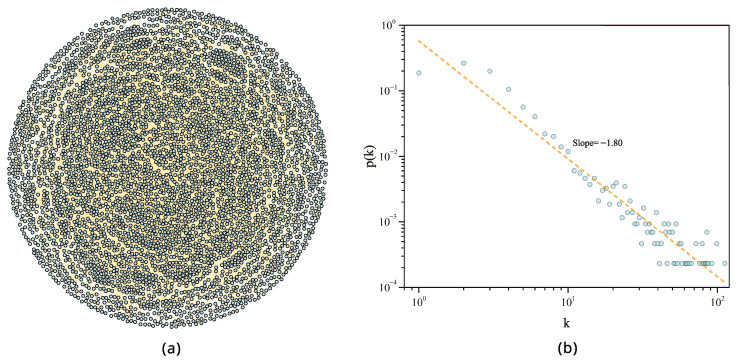
Topology of the P3SN. (**a**) Demonstration of the P3SN using the Fruchterman Reingold layout. (**b**) The degree distribution with power-law fit for the P3SN.

Based on the data of PPP programs, a shareholder network is constructed. A node denotes a shareholder, and if the *i*-th and *j*-th shareholders jointly participate in the same PPP program, an undirected link between the *i*-th node to the *j*-th node is drawn, as shown in Figure 2. Then, we construct an undirected and unweighted graph named “PPP shareholder network” (P3SN) shown in Figure 3a, containing 5944 nodes and 12,053 links. The giant connected component includes 4307 nodes and 10,627 links. The probability distribution of the degree is displayed in Figure 3b. We perform a power-law fit. However, it cannot pass the rigorous power-law test with the method in Ref. [40], although it is straight on a double-logarithmic scale. The rigorous scale-free structure is empirically rare across real-world networks [41]. However, the degree probability distribution is heavy-tailed, reflecting the typical non-equilibrium of complexity. In order to understand the topology and robustness of P3SN, we construct a random network for comparative analysis. As shown in Table 1, the average path length of P3SN is 5.504, which means that the flow of information or resources from a node to any other nodes needs to pass through five edges on average. The average path length of the random network is 5.437, which is close to that of P3SN. However, the average clustering coefficient of P3SN is 0.591, which is significantly larger than that of the random network. This is because the edges are formed between each enterprise participating in the same project. This implies that P3SN has a local financing cluster mode, and the P3SN is a small-world network.

## 4. Attack Strategy and Robustness

Network robustness describes the ability of a network to maintain its basic functions when it is subjected to interference or failures [42]. The robustness is not only for quantitatively unveiling the complex structure of the PPP system, but also practically for information dissemination and systematic risk. For the removal of nodes in random and in decreasing order of degree, the percolation process on networks has been carefully studied, and analytical results have been obtained in the limit of large network size (i.e., in the limit N→∞) [43,44]. Scale-free networks are robust against the random removal of nodes, but highly susceptible to targeted removal of degree. Here, we investigated the network robustness against targeted removal of nodes based on several non-local centrality measures rather than with a simple degree. It seems unrealistic to anticipate any analytical theory of the corresponding percolation process. Hence, we investigate it through computational methods.

### 4.1. Attack Strategy

This paper compares the targeted attack with the random attack, where the targeted attack refers to shareholder default, withdrawal, or bankruptcy, while the random attack refers to sudden policy changes or market fluctuations. In the targeted attack, nodes are removed in descending order based on one of the centrality measures, namely degree-based attack, entropy-based attack, betweenness-based attack, or closeness-based attack.

Following Refs. [34,45], two targeted attack strategies are adopted. We refer to them as initial targeted attack and update targeted attack in this paper. For the initial targeted attack, the centrality metrics of each node are calculated from the original network, and then sorted in descending order. The robustness indicators of the network are computed after a specified fraction of nodes are removed according to the order of node centralities. In contrast, for the update targeted attack, the centrality metrics of each node from the current network are recalculated and sorted after each removal. For the random attack, nodes are removed randomly, and the robustness indicators are averaged over 100 times. Finally, the robustness of the P3SN is measured by removing a given fraction of nodes.

Given the significant differences in node centrality values between the weighted and unweighted networks, the two attack strategies mentioned above can be further categorized into attacks based on weighted and unweighted key nodes. In this paper, we also construct a weighted P3SN, in which the weight of an edge between two nodes is defined as the number of projects they have co-participated in. To evaluate the necessity of distinguishing between weighted and unweighted key node attacks strategies, we compute the correlation coefficients between the rankings of their centrality metrics, as shown in Table 2. The high correlation of centrality rankings between weighted and unweighted P3SN implies that the attributes of nodes in P3SN depend primarily on the topological structure. Therefore, due to the high correlation of centrality rankings presented in Table 2, a robustness analysis based on the weighted network is omitted.

### 4.2. Robustness

**Figure 4:**
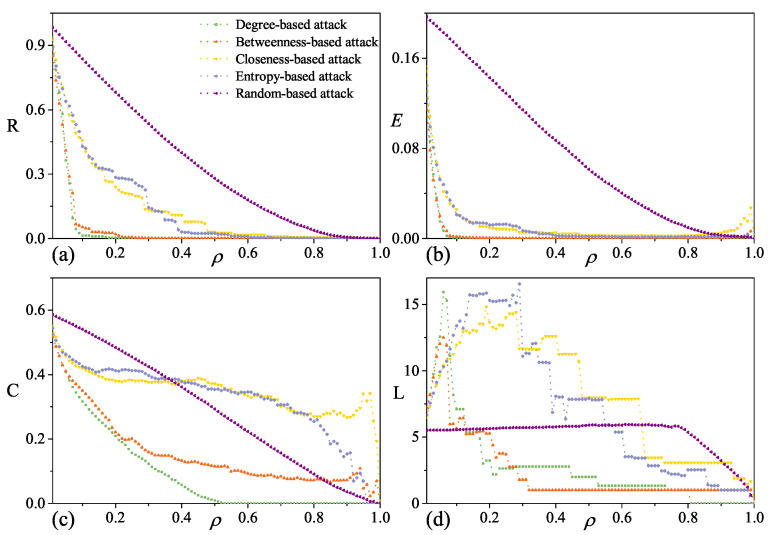
Robustness of P3SN under initial targeted attack. (**a**) Response behavior of relative size of largest component *R*, against six attack strategies. (**b**) Response behavior of network efficiency *E*. (**c**) Response behavior of average clustering coefficient *C*. (**d**) Response behavior of average path length *L*.

As displayed in Figure 4, different removal strategies have a significant impact on the topology of the P3SN network under initial targeted attack. Based on the decline rate of robustness indicators, attack strategies could be classified into three types: (1) degree-based and betweenness-based attack strategies, which lead to a rapid decline in network robustness; (2) closeness-based and entropy-based attack strategies, which exhibit a relatively gentle decline; and (3) random attack strategies, which show the slowest decline.

For both the targeted attack and random attack, *R* and *E* show similar decreasing behaviors. Under degree-based and betweenness-based attacks, they precipitously drop to zero after removing less than 10% of nodes. Moreover, *R* and *E* show exponential-like decay approaching zero after approximately 40% node removal under closeness-based and entropy-based attacks. *C* decreases slowly under the targeted attack, and the most efficient attack is the degree-based attack. After removing 40% of nodes, *C* declines more rapidly under the random attack than closeness-based and entropy-based attacks. Subsequently, *C* vanishes at around 50% node removal under the degree-based attack. That implies that the local financing cluster structure of P3SN is relatively stable. For the average path length, it increases as nodes are removed at first, and then decreases as network connectivity declines. *L* is destroyed after approximately 20% of nodes removal under the degree-based and betweenness-based attacks. In contrast, the random attack exhibits a completely different pattern of disruption.

**Figure 5:**
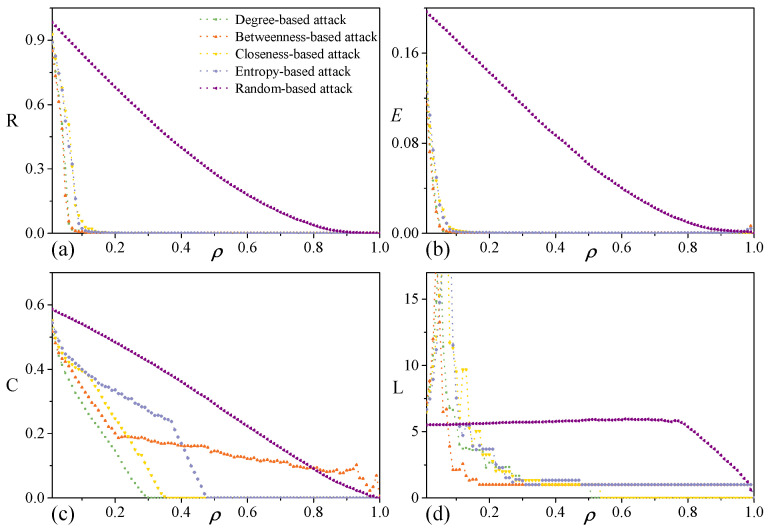
Robustness of P3SN under update targeted attack. (**a**) Response behavior of relative size of largest component *R*, against six attack strategies. (**b**) Response behavior of network efficiency *E*. (**c**) Response behavior of average clustering coefficient *C*. (**d**) Response behavior of average path length *L*.

As shown in Figure 5, under the update targeted attack, the classification of strategies based on R and E, while still present, becomes less distinct. *R* exhibits highly consistent values across different key node attack strategies, and this is particularly evident in the case of *E*. These two robustness metrics drop to zero after the removal of nodes less than 10%. Under the degree-based attack, the clustering coefficient C decreases most precipitously and drops to zero after 30% of nodes are removed. The closeness-based attack follows, with C falling to zero after approximately 35% of nodes are removed. L shows a sharp and interwoven decline pattern under four attacks compared to under the initial targeted attack, and it decreases most rapidly under the closeness-based attack.

**Figure 6:**
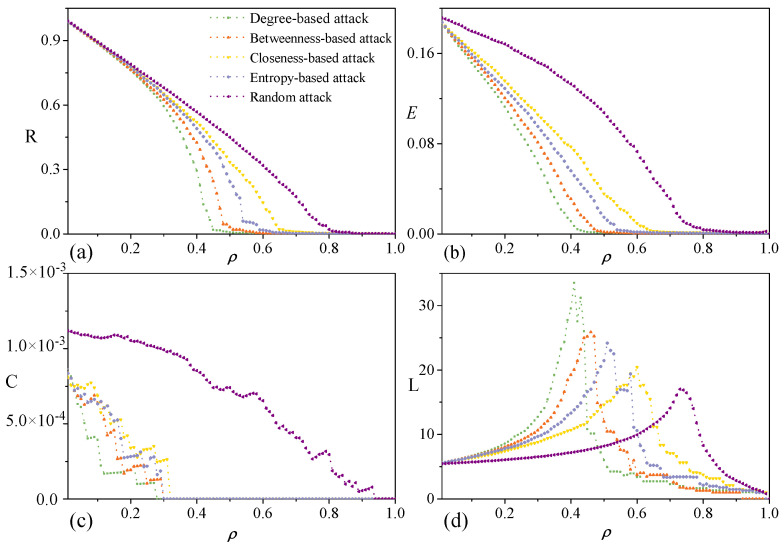
Robustness of random networks. (**a**) Response behavior of relative size of largest component *R*, against six attack strategies. (**b**) Response behavior of network efficiency *E*. (**c**) Response behavior of average clustering coefficient *C*. (**d**) Response behavior of average path length length *L*.

The robustness of the random network (Figure 6) reveals that classification phenomena similar to P3SN are absent. Quantitative comparison indicates that the attack strategies, ranked by their destructive efficiency, are as follows: degree attack being the most efficient, followed by betweenness-based attack, entropy-based attack, and closeness-based attack, with random attack being the least efficient. In addition, the degradation rates of all robustness indicators in the random network under targeted attack are significantly slower than those of real networks. Notably, for both the P3SN or random network, the degree-based attack is more effective than other attacks. This is consistent with the results in Ref. [34].

### 4.3. Comparison of Attack Strategies

As summarized in Table 3, different robustness metrics exhibit significant differences in their response to targeted attack strategies. Compared to the initial targeted attack, the updated targeted attack demonstrates significantly higher destructive efficiency, which is particularly evident under the closeness-based and entropy-based attacks. This result indicates that under the update targeted attack strategy, the network structure changes with each removal, which causes the sequence of key nodes to differ from that in the initial network. The P3SN exhibits superior robustness against the random attack compared to the targeted attack.

In addition, although the classification phenomenon is less distinct under the updated targeted attack, the threat posed by degree-based and betweenness-based attacks remains the highest. Notably, the classification pattern is entirely absent in the random network. Furthermore, under the targeted attack, the robustness metrics of the random network exhibited a slower decay rate than those of the P3SN. This indicates that the P3SN is more vulnerable to the targeted attack due to its heterogeneous and small-world structure.

## 5. Geographic Structure of Public–Private Partnership Networks

To investigate the geographic structure of the P3SN, shareholders are aggregated into cities with their registered company addresses. In particular, we define the shareholder map as a weighted graph, G(V,E,W), in which each node $v_{p}\in V$ corresponds to a city, $e_{pq}\in E$ is the edge between nodes *p* and *q*, and the weight of each edge $w_{pq}\in W$ corresponds to the total number of cooperation programs between any two shareholders registered in *p* to *q*. Thus, the weight of the edge reflects the strength between two cities.

Due to the dense connectivity of the original network, its key structural features are difficult to identify. In order to present the geographic structure of the P3SN more intuitively, this paper constructs a Minimum Spanning Tree (MST) based on the shareholder map. As an acyclic connected graph, MST can prioritize retaining important connections with higher weights while eliminating low-weight peripheral links, effectively extracting the core skeleton and hub nodes of the network [46]. Therefore, this article adopts the MST method to clearly reveal the backbone structure and key node distribution of the shareholder map.

The weight of edge is normalized as $w_{pq}^{\prime }=w_{pq}/\left(w_{max}\right)$, where $w_{max}$ is the maximal $w_{pq}$, and $w_{pq}^{\prime }=1$ if p=q. A metric distance between nodes $v_{p}$ and $v_{q}$ is defined by

(1) $$d_{pq}=\sqrt{2\left(1-w_{pq}^{\prime }\right)},$$

which forms an m×m distance matrix D. With the definition, $d_{pq}$ satisfies the three axioms of a metric (i) $d_{pq}=0$ if and only if p=q; (ii) $d_{pq}=d_{qp}$ and (iii) $d_{pq}\leq d_{pk}+d_{kq}$. The matrix D is then used to describe the MST connecting the *n* shareholders in the P3SN.

**Figure 7:**
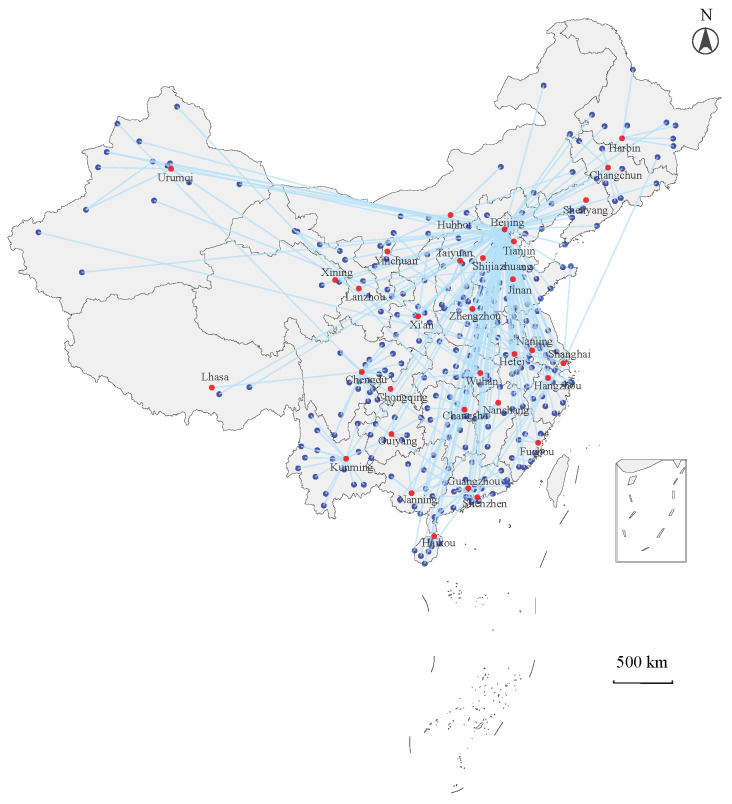
MST of the city network linked by P3SN. Red dots represent direct-administered municipalities and provincial capital. Blue dots represent other prefecture-level cities. Blue lines represent connections among different cities in MST.

As shown in Figure 7, the shareholder map of P3SN represents a typical hierarchical structure. The capital of China, Beijing, occupies the center of the map, and connects with other cities nationwide. Most provincial capitals occupy the sub-centers of the map, dominating local interactions in the province. This hierarchical structure visually confirms the role of Beijing as the central hub and the provincial capitals as regional centers.

**Figure 8:**
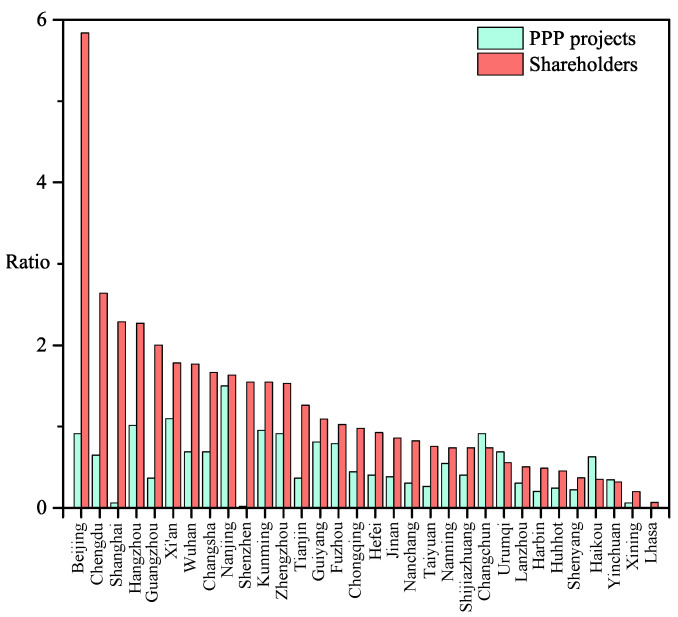
Geographic distribution of PPP projects and shareholders. Only direct-administered municipalities and provincial capitals are shown.

In addition, we calculate the percentages of PPP projects and shareholders located in direct-administered municipalities and provincial capitals. As shown in Figure 8, although Beijing hosts the largest number of shareholders, it has a relatively small number of projects. More notably, even though direct-administered municipalities and all provincial capitals own 40.17% of the total shareholders, they only own 17.19% of the PPP projects. This suggests that most PPP projects are located in small and medium-sized cities. However, considering the information communication and political operation, most shareholders are registered in direct-administered municipalities and provincial capitals.

## 6. Discussion

### 6.1. Key Nodes, Robustness, and Network Topology

**Figure 9:**
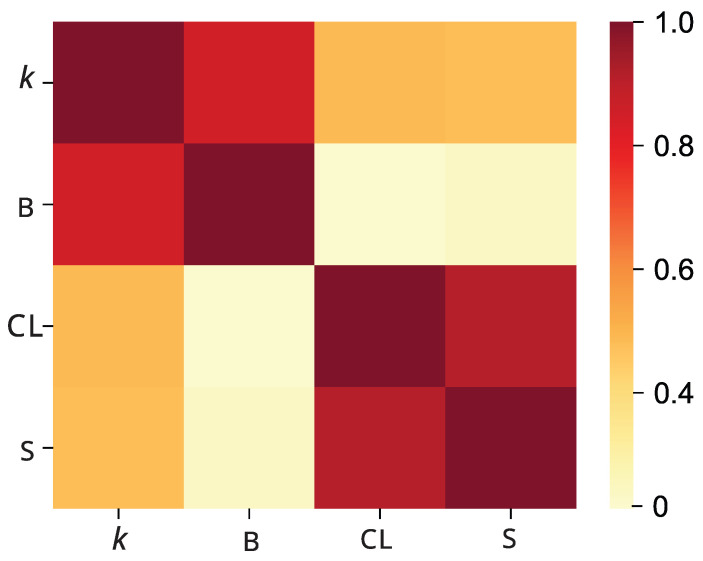
Heat map of Spearman correlation matrix for the P3SN.

Why can degree-based, betweenness-based, closeness-based, entropy-based, and random attacks be categorized into three types based on the robustness metric of P3SN? We compute the Spearman rank correlation coefficients between these centrality rankings of nodes. Figure 9 shows the heat map of the Spearman correlation matrix. The four centrality measures clearly separate into two distinct subgroups, i.e., degree and betweenness belong to one, while closeness and entropy are another. This suggests that high-degree nodes correlate with high betweenness, and high-closeness nodes have high entropy. This result is consistent with the classification of attack strategies. Similar classification results are also reported in Refs. [34,47], which analyze the robustness of scale-free networks and Boston gang network, respectively.

The results demonstrate that nodes with higher degrees act as a bridge role in connecting other nodes, as illustrated in Figure 2. If the shareholder network of each PPP project is considered as a subgraph, a node participating in multiple projects acts as a bridge connecting these subgraphs. The weighted degree centrality of this node corresponds to the total number of other nodes in these subgraphs, which explains why these nodes exhibit high degree and betweenness centralities. Thus, the removal of these nodes would lead to rapid network fragmentation and a sharp decline in network robustness. This implies that shareholders who frequently participate in projects connect shareholders from different projects. This suggests that these shareholders act as critical role in the flow of information and resources between projects, enabling rapid access network resources. Their failure can easily trigger a structural collapse of the network.

Within the P3SN, the weight of an edge denotes the number of projects co-participated by two shareholders. The high correlation of centrality rankings between weighted and unweighted P3SN implies that shareholders with high-weight edges participate in a multitude of projects, leading to relationships with a large number of shareholders. This suggests that shareholders prioritize the PPP project itself over the selection of partners when they make investment decisions.

The unique topological structure of the P3SN makes it not only with small-world characteristics and a heavy-tailed degree distribution, but also constitutes the intrinsic mechanism underlying its high sensitivity to the targeted attack. The heterogeneous connection pattern enhances the efficiency of information propagation, and leads to a few nodes with high centralities, which causes network vulnerability. Once shareholders with high centralities fall into risk, the robustness of the P3SN will be affected. Topological features of the P3SN enable efficient information transmission, while simultaneously introducing latent systemic risks.

### 6.2. Hierarchical Spatial Structure

PPP projects usually have characteristics such as large-scale investments, long operational cycles, and slow returns on investment, which poses high demands on abilities of private capital. Most key nodes of P3SN are often large enterprises located in direct-administered municipalities and provincial capitals, resulting in a hierarchical spatial structure. China State Construction Engineering Corporation Limited (CSCEC) serves as a typical example. As a core subsidiary of the central state-owned enterprises, it possesses substantial financial capital and an outstanding capacity for risk-bearing. According to data used in this paper, CSCEC has participated in over 70 PPP projects, establishing partnerships with well over 100 enterprises. This has solidified its position as one of the main hubs in the P3SN.

Network analysis also reveals a phenomenon of regional clustering in PPP collaborations. For example, within Shaanxi Province, CSCEC collaborates with local enterprises in cities such as Xi’an, Xianyang, and Weinan to participate in multiple local projects. This has thereby formed a regionally clustered cooperation network with Xi’an as its hub, characterized by partnerships between the central state-owned enterprise and local companies. However, as a leading enterprise located in Beijing, CSCEC also plays the crucial role of a “bridge” connecting these various regional clusters. Through its PPP project collaborations, its partners spread across more than 20 provinces, encompassing over 50 prefecture-level cities in China. This links multiple relatively independent regional networks into a nationwide financing cooperation system.

In the network of this study, shareholders in Beijing account for the largest proportion of the key nodes, reaching over 30%. The remaining key nodes are almost located in direct-administered municipalities and provincial capital. Through their participation in multiple PPP projects and collaborations with shareholders across the country, Beijing serves as a global hub connecting regional clusters throughout the country, and direct-administered municipalities and provincial capitals act as local hubs. The key nodes located in these hubs possess the ability to integrate resource across regions.

### 6.3. Network Evolution

We construct networks from 2012 to 2019, as shown in Table 4. The network evolution can be divided into three stages. The first stage, prior to 2015, represents the exploration stage of the PPP mode. The number of PPP projects was less than 200, and the scale of the network was relatively small, containing nodes less than 30 and links less than 100. This resulted in a short average path length (L<2.5). Although *C* was less than 0.500 in 2012 and 2013, it reached 0.720 in 2014, indicating that clusters have formed in P3SN. The second stage, from 2015 to 2017, marks the promotion stage of the PPP mode. Since 2014, a series of policies about PPP mode were issued in China. The number of PPP projects surged from 169 in 2014 to 1283 in 2015. As the number increased and new shareholders joined, the scale of P3SN began to expand and became sparser. *L* increased from 2.407 to 5.960, while *C* decreased from 0.720 to 0.647. The clustered structure still existed in P3SN. As the number of PPP projects increased, problems of PPP mode began to emerge. Since 2017, China has issued more policies concerning management and regulation of PPP mode. These policies standardized the development of PPP mode, making PPP mode move towards a stable development stage (from 2018 to 2019). L and C became convergent around 5.5 and 0.59, respectively.

The three stages of PPP mode development demonstrate that the evolution of P3SN is driven significantly by policies in China. In comparison, the network of PPP sponsors in European countries (e.g., Germany, Spain, France, the Netherlands, Italy, and the United Kingdom) also exhibits small-world and clustering characteristics [48]. In addition, similar to P3SN of China, a small number of nodes with high centralities connect the clusters of the network. In Canada and Chile, the formation of a homogeneous PPP equity network is driven by central governments. However, in the US, state governments contribute to generate a heterogeneous PPP network [49].

### 6.4. Implications

This study has significant importance for regulatory authorities for the identification of key enterprises and projects, thereby enabling targeted interventions and enhanced oversight. In order to improve the robustness of P3SN, regulatory authorities should focus on the enterprises identified as systemically important nodes (e.g., top 10% by centrality) in the P3SN. This requires special supervision on enterprises with high degree or betweenness centrality, which act as critical hubs. Furthermore, the PPP projects related to these enterprises should also be rigorously monitored to prevent localized risks from these critical hubs.

This study also leaves some questions for future research. First, how do various risk types impact the shareholder network? In particular, what are the transmission paths and the scope of their impact? Second, what is the correlation between the spatial distribution of enterprises, projects, and regional economic-geographic factors? Third, how does the topology (e.g., centrality, and geographic structure) and robustness of the P3SN evolve dynamically? Investigating these can provide information for the formulation of PPP policies in the region.

## 7. Conclusions

This study systematically reveals the structural complexity, robustness, and geographical structure of PPP shareholder collaboration networks in China. The P3SN exhibits a small-world structure. Attacks for the P3SN show that degree-based and betweenness-based attacks are of the same class, entropy-based and closeness-based attacks belong to another, while random attack behaves separately. By comparison, the robustness of the P3SN is weaker than that of the random network, especially under the targeted attack. Analysis of the correlation between network topology and robustness reveals that, in practice, the withdrawal of key enterprises from PPP projects will result in a significant impact on the stability of the P3SN. The geographic structure of the P3SN shows that Beijing dominates the PPP system globally, and provincial capitals occupy sub-center of the network.

Based on above, administrative departments should focus on key enterprises, especially the leading enterprises that occupy the dominant position in the region. In addition, it is also necessary to strengthen the risk monitoring of PPP projects involving those key nodes enterprises. Furthermore, when risk emerges in key enterprises, risk prevention should be established to effectively prevent risk propagation.

## Figures and Tables

**Table 1:** The properties of the P3SN and random network. *N*, *M*, *k*, *L*, and *C* represent the number of nodes, the number of links, the average degree, the average path length, and the average clustering coefficient, respectively.

Network	*N*	*M*	*k*	*L*	*C*
P3SN	4307	10,627	4.935	5.504	0.591
Random	4307	10,627	4.935	5.437	0.001

**Table 2:** The correlation coefficients between the rankings of centrality metrics of weighted and unweighted P3SN. *r* represents the correlation coefficients.

Centrality	Degree	Betweenness	Closeness	Entropy
*r*	0.986	0.985	0.962	0.99

**Table 3:** The robustness results of P3SN.

Robustness Metrics	Initial Targeted Attack	Update Targeted Attack
R	R declines most rapidly under degree-based and betweenness-based attacks, with the network completely collapsing after approximately 10% of nodes are removed.	The classification phenomenon is less evident relative to the initial targeted attack. R drops to zero under the targeted attack within 10% nodes removed.
E	E declines more rapidly than R under closeness-based and entropy-based attacks.	The behavior of E is similar to that of R, but it declines even more rapidly.
C	Under degree-based attack, C declines most rapidly and drops to zero after approximately 50% of nodes are removed.	C declines most rapidly under degree-based attack, and drops to zero about 30% nodes removed.
L	L is destroyed after 20% nodes removed under degree-based and betweenness-based attacks.	L shows a sharp and interwoven decline pattern compared to under the initial targeted attack, and it decreases most rapidly under the closeness-based attack.

**Table 4:** Annual topological metrics of the P3SN from 2012 to 2019. *N*, *M*, *k*, *L*, *C*, and *P* represent the number of nodes, the number of links, the average degree, the average path length and the average clustering coefficient, and the cumulative number of PPP projects, respectively.

Year	*N*	*M*	*k*	*L*	*C*	*P*
2012	7	10	2.857	1.667	0.486	14
2013	8	11	2.750	1.821	0.404	39
2014	25	71	5.680	2.407	0.720	169
2015	882	2344	5.315	5.960	0.647	1283
2016	2356	5557	4.717	5.601	0.596	2843
2017	3961	9631	4.863	5.580	0.589	4516
2018	4272	10,464	4.899	5.506	0.591	4870
2019	4307	10,627	4.935	5.504	0.591	4933

## Data Availability

Restrictions apply to the availability of these data. Data were obtained from the Wind Financial Database and are available via institutional subscription.
